# Sensorineural hearing loss induced by gefitinib: A CARE-compliant case report and literature reviews

**DOI:** 10.1097/MD.0000000000036010

**Published:** 2023-11-10

**Authors:** Bao-chen Zhu, Wen-hua Yang, Mao Huang, Jin-gui Wang, Yan Liang, Zhen-zhen Lei, Sha-sha Zhang, Ying Wang, Xiao-di Sun, Ying Gong, Chun-miao Xue, Guo-dong Hua

**Affiliations:** a Department of Pharmacy, Dongzhimen Hospital, Beijing University of Chinese Medicine, Beijing, China; b Department of Respiratory, Dongzhimen Hospital, Beijing University of Chinese Medicine, Beijing, China; c Department of Pharmacy, Dongfang Hospital, Beijing University of Chinese Medicine, Beijing, China.

**Keywords:** gefitinib, EGFR-TKI, sensorineural hearing loss, adverse drug reaction

## Abstract

**Rationale:**

Gefitinib is a potent and selective orally active growth factor receptor (EGFR)-tyrosine kinase inhibitor that is commonly used to treat advanced non-small cell lung cancer patients with activating EGFR mutations. Hearing impairment with gefitinib was sparsely reported. In this report, we describe a case of sensorineural deafness associated with the administration of gefitinib, with a Naranjo score of 7.

**Patient concerns:**

An 81-year-old female was diagnosed with lung adenocarcinoma with bone metastasis and an EGFR-activating mutation. The patient was prescribed gefitinib tablets at a daily dose of 250 mg for lung adenocarcinoma treatment. However, the patient experienced moderate to severe bilateral sensorineural deafness, primarily in her right ear, after taking gefitinib. Following the cessation of gefitinib administration, the patient exhibited partial restoration of auditory function. Upon resuming the medication, she experienced a worsening of deafness.

**Diagnoses:**

The otoscopic audiogram and hearing test indicated moderate to severe bilateral sensorineural deafness.

**Interventions:**

The otolaryngologist recommended bilateral hearing aids to enhance hearing function.

**Outcomes:**

Throughout our follow-up period, the patient did not receive a hearing aid implant.

**Lessons:**

This article first reported the ototoxicity caused by gefitinib. While rare, our report highlights that gefitinib-induced sensorineural deafness is possible and its mechanisms are still unclear. This adverse reaction should be monitored closely during clinical application of gefitinib to improve patient outcomes.

## 1. Introduction

Gefitinib is a novel targeted therapeutic agent that acts as a selective epidermal growth factor receptor tyrosine kinase inhibitor (EGFR-TKI) for the treatment of patients with locally advanced or metastatic non-small cell lung cancer with epidermal growth factor receptor gene (EGFR) sensitive mutations. It selectively interferes with EGFR receptor tyrosine kinase activity by competing with adenosine triphosphate for the EGFR binding site, thereby inhibiting tumor neovascularization, blocking blood supply to tumor cells, and inhibiting mitogen-activated protein kinase activation and promoting apoptosis.^[1,2]^

Multiple randomized controlled studies have shown that first-generation selective EGFR-TKI represented by gefitinib can significantly improve the progression-free survival time of patients with advanced EGFR-mutation-positive non-small cell lung cancer compared with chemotherapy, and the adverse reactions of grade 3 and above are significantly lower than those of chemotherapy. Thus EGFR-TKI is first-line treatment in patients with advanced EGFR-mutation-positive non-small cell lung cancer.^[3]^ The most common adverse reactions of gefitinib mainly involve the cutaneous system (rash), digestive system (diarrhea), and hepatobiliary system (elevated transaminase).^[4,5]^ In recent years, reports of severe adverse reactions of gefitinib in literature mainly include: interstitial lung disease,^[6]^ fulminant purpura,^[7]^ acute cholecystitis,^[8]^ bone marrow suppression,^[9]^ severe diarrhea,^[10]^ retinal hemorrhage,^[11]^ etc. Herein, we report a case of acute sensorineural deafness after the administration of gefitinib in a patient with lung adenocarcinoma. At the same time, the literature on hearing impairment caused by EGFR-TKI was summarized, so as to provide reference for clinical use of EGFR-TKI. Ethics Committee of Dongzhimen Hospital Affiliated to Beijing University of Chinese Medicine approved the study, and an informed consent has provided.

## 2. Details of the case

The patient in question was an 81-year-old female who presented to our clinic with cough, sputum production, shortness of breath, and decreased activity endurance with no apparent cause. A computed tomography scan revealed a right lung mass, a right upper lung nodule shadow, enlargement of 2 lung hilar shadows, right-sided massive pleural effusion associated with pulmonary atelectasis and incomplete expansion, interstitial changes in both lungs, and multiple tiny nodular shadows. The pleural fluid pathology report indicated adenocarcinoma, while the results of gene testing confirmed an EGFR-activating mutation. The patient was therefore diagnosed with lung adenocarcinoma with bone metastasis and was placed on gefitinib tablets at a dose of 250 mg once daily, beginning on May 15, 2020. The patient also had a preexisting medical history of hypertension, hyperlipidemia, and coronary atherosclerotic heart disease and was receiving regular symptomatic treatment with valsartan, amlodipine, simvastatin, ezetimibe, aspirin, isosorbide mononitrate, and trimetazidine hydrochloride.

On October 13, 2020, the patient’s cough and wheezing symptoms worsened and she was admitted to the hospital. The patient presented with a complaint of drug-induced hearing loss following a 4-month duration of gefitinib administration. As a consequence, the patient made an independent decision to discontinue the medication on September 30, resulting in a partial recovery of her auditory function. Despite this improvement, the patient’s cough and asthma symptoms persisted, leading to the resumption of gefitinib treatment as advised by her attending physician. However, after taking gefitinib for 3 days, she reported a worsened deafness, particularly in her right ear. The otoscopic audiogram and hearing test indicated moderate to severe bilateral sensorineural deafness. The otolaryngologist recommended bilateral hearing aids to enhance hearing function. However, throughout our follow-up period, the patient did not receive a hearing aid implant. Meanwhile, her hearing loss did not alleviate during the continuous administration of gefitinib, persisting until her passing.

## 3. Discussion

The patient had no history of deafness or hearing loss before taking gefitinib, but hearing loss occurred about 4 months after taking gefitinib. During gefitinib administration, the drugs used were commonly used for previous underlying diseases, and no other drugs were used. The patient’s deafness symptoms relieved after stopping gefitinib, but worsened after resuming the medication. The Naranjo Scale Questionnaire,^[12]^ which was developed to standardize the causality assessment of adverse drug reaction, was used to estimate the probability of adverse drug reaction in this case. It was found the final score to be 7, indicating the correlation between sensorineural deafness and gefitinib use was determined as “probable,” that is, supported by objective evidence or quantitative test results. So the clinical pharmacist reported this case of adverse reaction to National adverse drug reaction surveillance system of China.

Sensorineural deafness is an organic disease of Corti’s organ, auditory nerve and auditory center, which leads to various types of hearing loss or loss due to its obstruction of sound perception and analysis or influence of information transmission. There are many causes of sensorineural deafness, including age, noise, heredity and the use of ototoxic drugs.

Gefitinib is a selective EGFR TKI. There are only a few reports on the ototoxicity of EGFR-TKI in recent years. Yet, the underlying mechanism is incompletely understood. Some studies showed that it may be related to the expression of EGFR receptor in the inner ear.^[13]^ EGFR receptor is expressed in both sensory and non-sensory cells of the inner ear of newborn/adult mice, and is involved in the survival, proliferation, regeneration, synaptic connection, cochlear balance and other processes of inner ear cells.^[14]^ Corti’s organ is an important part of sound perception and is composed of sertoli cells and hair cells, etc. Each hair cell forms a synaptic connection with nerve fibers, and the loss of synaptic conduction between hair cells and sensory neurons is a major factor of sensorineural hearing loss.^[15]^ Studies have found that EGFR receptor is related to the response of rat cochlear Corti’s organ to ototoxic drug.^[16]^ Furthermore, transgenic mice blocked by EGFR signal transduction pathway may produce severe hearing loss.^[17]^ Therefore, EGFR-TKI may affect multiple processes of nerve cells in the inner ear by inhibiting EGFR signaling pathway, and resulting in ototoxicity. However, this mechanism has been rarely reported in the clinical use of EGFR-TKIs. Search through the literature has yielded only 1 report of EGFR-TKI-induced hearing impairment, wherein a 66-year-old patient developed tinnitus, dizziness, and profound bilateral hearing loss within 0.5 hour after administration of 150 mg of oral erlotinib, leading to irrevocable ototoxicity. However, Gefitinib-induced hearing loss has not been reported in the literature.

The elderly patient in this case had a previous history of high blood pressure, hyperlipidemia, and coronary atherosclerotic heart disease. She regularly uses Valsartan, amlodipine, simvastatin, ezetimibe, aspirin, isosorbide mononitrate, and trimetazidine hydrochloride, which are not mentioned as having the potential for hearing impairment in the dispensatory. Gefitinib is mainly metabolized by CYP3A4 and may interact with CYP3A4 inducers, inhibitors, or substrates. Simvastatin and amlodipine, which are substrates of CYP3A4 among the patient’s common medications, may compete with gefitinib for the same metabolic pathway and increase the blood concentration of gefitinib, which may be one of the causes of hearing loss in the patient. This is also a limitation of our study. Due to the multitude of co-administered medications, it is unclear whether the sensorineural hearing loss induced by gefitinib is solely due to the drug’s individual adverse reaction or if it is related to interactions with another medication taken by the patient.

## 4. Conclusion

This article is the first to report on the ototoxicity of gefitinib. It should be emphasized that gefitinib dosage may lead to irreversible damage to hearing, reinforcing the need for diligent monitoring of patients who are prescribed this medication. Audiometric screening at regular intervals is an essential component of patient care in these instances.

## Author contributions

**Data curation:** Bao-chen Zhu, Wen-hua Yang, Mao Huang, Yan Liang, Zhen-zhen Lei, Sha-sha Zhang, Ying Wang, Xiao-di Sun.

**Supervision:** Ying Gong, Chun-miao Xue, Guo-dong Hua.

**Writing – original draft:** Bao-chen Zhu, Wen-hua Yang.

**Writing – review & editing:** Jin-gui Wang.

## References

[1] Ma Y Xin S Huang M. Determinants of gefitinib toxicity in advanced non-small cell lung cancer (NSCLC): a pharmaco-genomic study of metabolic enzymes and transporters. Pharmacogenomics J. 2017;17:325–30. 27089937 10.1038/tpj.2016.31

[2] Zhu C Cai ZA Li XY. Clinical study of gefitinib combined with concurrent thoracic radiotherapy in the treatment of locally advanced non-small cell lung cancer with sensitive gene mutation (in Chinese). China Basic Med. 2019;26:943–8.

[3] Chinese Society of Lung Cancer, Chinese Anti-Cancer Association. EGFR-TKI ADR management Chinese expert consensus (in Chinese). Chin J Lung Cancer. 2019;22:57–81. 10.3779/j.issn.1009-3419.2019.02.01 PMC6397940 30827323

[4] Xia HH. Literature analysis of 82 cases of adverse drug reactions induced by Gefitinib (in Chinese). Chinese J Pharmacovigilance. 2016;13:98–102.

[5] Cheng H Liu H Du Q. Efficacy and safety of domestic and imported gefitinib in patients with advanced non-small cell lung cancer. Ann Palliat Med. 2021;10:10–5. 33440959 10.21037/apm-20-2140

[6] Beom SH Kim DW Sim SH. Gefitinib-induced interstitial lung disease in Korean Lung cancer patients. Cancer Res Treat. 2016;48:88–97. 25761482 10.4143/crt.2014.201 PMC4720097

[7] Fan PL Lu LL Wang YM. Analysis of one case of purpura fulminans induced by gefitinib tablets (in Chinese). Chinese J Pharmacovigilance. 2020;17:441–3.

[8] Liao YF Yue JN Si K. One case of severe serum transaminase elevation with acute cholecystitis induced by gefitinib tablets (in Chinese). Chinese J Pharmacovigilance. 2021;18:584–7.

[9] Sun LE Huang YX Wang J. One case of grade IV myelosuppression caused by gefitinib (in Chinese). Chin J Mod Appl Phar. 2021;38:1608–9.

[10] Yang G Xu W. Death due to sever diarrhea with electrolyte disturbance and gastrointestinal hemorrhage induced by gefitininb (in Chinese). ADR J. 2019;21:473–4.

[11] Gu H Jiang HD. Retinal hemorrhage caused by gefitinib (in Chinese). ADR J. 2021;23:52–3.

[12] Naranjo CA Busto U Sellers Edward M. A method for estimating the probability of adverse drug reactions. Clin Pharmacol Ther. 1981;30:239245. 10.1038/clpt.1981.154 7249508

[13] Koutras AK Mastronikolis NS Evans TR. Irreversible ototoxicity associated with the use of erlotinib in a patient with pancreatic cancer. Acta Oncol. 2008;47:1171–3. 18615326 10.1080/02841860802213328

[14] Hume CR Kirkegaard M Oesterle EC. ErbB expression: the mouse inner ear and maturation of the mitogenic response to heregulin. J Assoc Res Otolaryngol. 2003;4:422–43. 14690060 10.1007/s10162-002-3008-8 PMC3202727

[15] Qin XM Qin XG. Research status and treatment progress of sensorineural deafness loss (in Chinese). Chinese Manipulation Rehab Med. 2021;12:73–5.

[16] Zine A Nyffeler M de Ribaupierre F. Spatial expression patterns of epidermal growth factor receptor gene transcripts in the postnatal mammalian cochlea. Hear Res. 2000;141:19–27. 10713492 10.1016/s0378-5955(99)00203-8

[17] White PM Stone JS Groves AK. EGFR signaling is required for regenerative proliferation in the cochlea: conservation in birds and mammals. Dev Biol. 2012;363:191–200. 22230616 10.1016/j.ydbio.2011.12.035 PMC3288574

